# Abnormalities in gray matter volume in patients with borderline personality disorder and their relation to lifetime depression: A VBM study

**DOI:** 10.1371/journal.pone.0191946

**Published:** 2018-02-21

**Authors:** Salvatore Aguilar-Ortiz, Pilar Salgado-Pineda, Josep Marco-Pallarés, Juan C. Pascual, Daniel Vega, Joaquim Soler, Cristina Brunel, Ana Martin-Blanco, Angel Soto, Joan Ribas, Teresa Maristany, Salvador Sarró, Raymond Salvador, Antoni Rodríguez-Fornells, Edith Pomarol-Clotet, Peter J. McKenna

**Affiliations:** 1 FIDMAG Germanes Hospitalàries Research Foundation, Barcelona, Spain; 2 Benito Menni Centre Assistencial en Salut Mental, Sant Boi de Llobregat, Barcelona, Spain; 3 Departament de Psiquiatria i Medicina Legal. PhD Programme, Doctorat de Psiquiatria. Universitat Autònoma de Barcelona (UAB), Barcelona, Spain; 4 CIBERSAM, Barcelona, Spain; 5 Faculty of Psychology, University of Barcelona, Bellvitge Hospital, Barcelona Spain; 6 Department of Psychiatry, Hospital de la Santa Creu i Sant Pau, Barcelona Spain; 7 Servei de Psiquiatria i Salut Mental, Consorci Sanitari de l’Anoia, Igualada Spain; 8 Hospital Sant Joan de Déu, Esplugues de Llobregrat, Spain; Public Library of Science, UNITED KINGDOM

## Abstract

**Background:**

Structural imaging studies of borderline personality disorder (BPD) have found regions of reduced cortical volume, but these have varied considerably across studies. Reduced hippocampus and amygdala volume have also been a regular finding in studies using conventional volumetric measurement. How far comorbid major depression, which is common in BPD and can also affect in brain structure, influences the findings is not clear.

**Methods:**

Seventy-six women with BPD and 76 matched controls were examined using whole-brain voxel-based morphometry (VBM). The hippocampus and amygdala were also measured, using both conventional volume measurement and VBM within a mask restricted to these two subcortical structures. Lifetime history of major depression was assessed using structured psychiatric interview.

**Results:**

At a threshold of p = 0.05 corrected, the BPD patients showed clusters of volume reduction in the dorsolateral prefrontal cortex bilaterally and in the pregenual/subgenual medial frontal cortex. There was no evidence of volume reductions in the hippocampus or amygdala, either on conventional volumetry or using VBM masked to these regions. Instead there was evidence of right-sided enlargement of these structures. No significant structural differences were found between patients with and without lifetime major depression.

**Conclusions:**

According to this study, BPD is characterized by a restricted pattern of cortical volume reduction involving the dorsolateral frontal cortex and the medial frontal cortex, both areas of potential relevance for the clinical features of the disorder. Previous findings concerning reduced hippocampus and amygdala volume in the disorder are not supported. Brain structural findings in BPD do not appear to be explainable on the basis of history of associated lifetime major depression.

## Introduction

Borderline personality disorder (BPD) is characterized by identity disturbance, unstable and intense interpersonal relationships, impulsive and self-damaging behavior, anger dyscontrol, affective instability, problems tolerating being alone, and chronic feelings of emptiness [1,2]. Transient and stress-related psychosis-like manifestations are also a recognized feature of the disorder [3] and a proportion of patients have more enduring psychotic symptoms, especially hallucinations [4,5]. A clinical association with major affective disorder is increasingly recognized [6–8], with up to 80% of patients meeting criteria for major depressive disorder at some point in their lives [9]; and approximately 20% meeting criteria for bipolar I or II disorder [10]. A link with post-traumatic stress disorder has been proposed but is currently not unequivocally established [11].

Biological factors in BPD are a focus of considerable current interest, with genetic, neuroendocrine and brain imaging studies all reporting positive findings [2,12,13]. Structural imaging studies in particular have implicated the amygdala and hippocampus, with reduced volume of these structures being found in two meta-analyses [14,15]. Cortical volume reductions have also been found in studies examining selected regions of interest (ROIs), including in the anterior cingulate cortex, the orbitofrontal cortex and the right parietal cortex [16–19].

Around 10 studies to date have also examined brain structure in BPD using voxel-based morphometry (VBM) [20]. This technique has advantages over conventional brain volume measurement in that it examines across the whole brain and so can detect changes that may be small and/or do not correspond to accepted anatomical subdivisions. The majority of these studies have been small (range 7–34 patients and corresponding numbers of controls), although two recent studies compared 68 patients and 52 controls [21] and 60 patients and 60 controls [22], respectively. A meta-analysis of these studies [23] found support for volume reductions in the pars opercularis and triangularis of the right inferior frontal gyrus, and in parts of the temporal lobe cortex bilaterally, and less robustly in the precentral gyrus and the right superior frontal gyrus. Volume increases were found in the right cerebellum, the right supplementary motor area, the left rolandic operculum and the right middle frontal gyrus. This meta-analysis also found pooled evidence for volume reductions in the right hippocampus, and less robustly in the left hippocampus using VBM.

Both major depression [24,25] and bipolar disorder [26–28], are known to be associated with cortical volume reductions. Reduced volume of the hippocampus is also a feature of major depression [24], and has recently been found in bipolar disorder as well in a large study [29].

Nevertheless, whether this is a factor relevant to the brain structural findings in BPD has been examined in relatively few studies, either using conventional volumetric measurement [16,17,30,31] or VBM [32,33]. Nor was history of affective disorder examined as a moderating factor in the above meta-analysis of voxel-based studies [23].

In the present study we aimed to evaluate brain structural abnormalities using VBM and whole-brain analysis in a relatively large sample of patients with BPD and well-matched healthy controls. We examined hippocampal and amygdala volume using both voxel-based and conventional measurement techniques. Additionally, we considered the question of to what extent any brain structural differences from controls found might be related to history of major depressive disorder in the patients.

## Material and methods

### Subjects

Seventy-six female out-patients with BPD were recruited from two hospitals in Barcelona with specialized units for the disorder: Hospital de la Santa Creu i Sant Pau and the Consorci Sanitari de l’Anoia. Participants were recruited from December 2010 to September 2011. Although a small number of men were also seen over this period, it was decided not included them in order to avoid the potential noise due to normal gender differences in brain structure [34].

Inclusion criteria were: 1) diagnosis of BPD made according DSM-IV criteria; this was made on the basis of the Spanish version of the Structured Clinical Interview for DSM-IV Axis II Personality Disorders (SCID II) [35]; patients were also required to score above the cutoff (score ≥ 6) on the Spanish Revised Diagnostic Interview for Borderlines [36] (DIB-R). 2) age between 18 and 55 years, 3) right-handed; and 4) having an IQ in the normal range (based on available clinical information plus an IQ-estimate based on two subsets of the WAIS-III: Vocabulary and Matrix Reasoning).

Exclusion criteria included: IQ under 70, a history of brain trauma (as indexed by skull fracture, loss of consciousness for more than 24 hours or presence of post-traumatic amnesia) or neurological disease, or having shown alcohol/substance abuse or dependence within the six months prior to participation. Patients were assessed for presence of other mental disorders, specifically schizophrenia, schizoaffective disorder, bipolar disorder and major depressive disorder,using the lifetime version of the Structured Clinical Interview for DSM-IV (SCID-I) [37]. Any who had a history of schizophrenia, schizoaffective disorder or bipolar disorder were excluded. Those with a history of major depressive disorder were not excluded, but presence of such episodes was recorded (see below). Patients with current major depression were excluded.

Forty-one of the patients were on psychotropic medication. Twenty-one were taking antidepressants (12 with adjunctive mood stabilizer, 9 without mood stabilizer). Four patients were taking antipsychotics (2 with adjunctive mood stabilizer, 2 without mood stabilizer). Seven patients were treated with both, antidepressants and antipsychotics (6 with adjunctive mood stabilizer, 1 without mood stabilizer). Seven patients were on mood stabilizers alone.

The control sample consisted of 76 right-handed healthy women selected to be age and IQ-matched to the patients. They were recruited from non-medical staff working in a hospital (Benito Menni CASM), their relatives and acquaintances, and also from sources in the community who were recruited by advertisement, and word of mouth. They met the same exclusion criteria as the BPD group. They were interviewed by a clinical psychologist or psychiatrist and were excluded if they reported a history of any psychiatric disorder or were taking any type of psychotropic medication other than non-regular use of benzodiazepines or other similar drugs for insomnia. They were also questioned about family history of mental illness and excluded if a first-degree relative had experienced symptoms consistent with major psychiatric disorder.

The study was carried out in accordance with the latest version of the Declaration of Helsinki and was approved by the relevant research ethics committee (Comité Ético de Investigación Clínica de las Hermanas Hospitalarias, Barcelona). Written informed consent was obtained for all subjects. The participants did not receive any economic compensation.

### Clinical assessment

All patients were assessed by clinical psychologists who were experienced in the evaluation and treatment of BPD. Symptom measures included: 1) The Structured Clinical Interview for DSM-IV Axis II Personality Disorders (SCID-II) [35,38]. 2) The Spanish version of the Revised Diagnostic Interview for Borderlines (DIB-R) [36]. This is a semi-structured interview, which assesses features of BPD over the last two years. The Spanish version has shown good psychometric properties regarding internal consistency (Cronbach’s alpha: 0.89), inter-rater reliability (within-class correlation: 0.94), sensitivity (0.81), and specificity (0.94). 3) The Spanish version of Borderline Symptom List– 23 (BSL-23) [39,40]. This is a self-rating instrument used to assess the type and severity of symptoms seen in BPD. It represents a shortened form of BSL-95, and includes 23 items from the original 95 that have high sensitivity to change and high ability to discriminate BPD from other pathologies. We used a validated Spanish version with good psychometric properties in terms of total internal consistency (Cronbach’s alpha: 0.936), one-week temporal reliability (r = 0.73), correlation with DIB-R scores and discrimination among different levels of BPD severity. For technical reasons it was only possible to carry out the BSL-23 on 54 patients.

Presence or absence of comorbid major depressive disorder was established from the lifetime SCID-I [37].

### Image acquisition and pre-processing

All subjects underwent a single MRI scanning session using a 1.5 Tesla GE Signa scanner (General Electric Medical Systems, Milwaukee, Wisconsin) located at the Sant Joan de Déu Hospital in Barcelona (Spain). High resolution structural T1 MRI data were acquired with the following acquisition parameters: matrix size 512x512; 180 contiguous axial slices; voxel resolution 0.47x0.47x1 mm3; echo (TE), repetition (TR) and inversion (TI) times, (TE/TR/TI) = 3.93 ms/2000 ms/710 ms respectively; flip angle 15 degrees.

### Whole-brain analysis

Structural data was analyzed with FSL-VBM [41], http://fsl.fmrib.ox.ac.uk/fsl/fslwiki/FSLVBM), an optimized VBM protocol [42] carried out with FSL tools [43]. This method takes TIV into account. First, structural images were brain-extracted and gray matter-segmented before being registered to the MNI 152 standard space using non-linear registration [44]. The resulting images were averaged and flipped along the x-axis to create a left-right symmetric, study-specific gray matter template. Secondly, native gray matter images were non-linearly registered to this study-specific template and ‘modulated’ to correct for local expansion (or contraction) due to the non-linear component of the spatial transformation. The modulated gray matter images were then smoothed with an isotropic Gaussian kernel with a sigma of 4 mm. Finally, voxelwise GLM was applied using permutation-based non-parametric testing, correcting across space using the ‘threshold-free cluster enhancement’ (TCFE) method [45].

Group comparison of gray matter VBM images was carried out by fitting general linear models. Statistical significance was assessed through permutation-based non-parametric tests (the randomize function in FSL) [46]. Correction for multiple comparisons was performed using a TCFE FWE-corrected p-value of 0.05.

ROIs based on clusters where there were significant differences between the patients and the controls in the whole brain analysis were extracted and used to examine associations with clinical features (severity of borderline symptomatology and history of major depressive disorder).

### Analysis of hippocampal and amygdala volume

To compare hippocampal and amygdala volumes between groups we defined ROIs for these two structures using the Harvard-Oxford Subcortical atlas provided in the FSL package. Mean volumes for each subject in these ROIs were extracted from the individual GM-maps using FIRST [47], a model-based segmentation/registration tool part of FSL [43] that measures the volume of thalamus, caudate nucleus, putamen, pallidum, nucleus accumbens, hippocampus and amygdala. Volumes were calculated for the left and right hippocampal and amygdala separately and compared between groups using two-tailed t-test to test for differences. Results are presented uncorrected and also corrected for multiple comparisons; given that the volumes would be expected to be independent of each other, Benjamini and Yekutieli’s False Discivery Rate (FDR) for non-independent variables [48] is more appropriate than Bonferroni correction here.

As well as absolute volumes, normalized volumes (i.e. absolute volume divided by overall brain volume) were calculated. Normalized volumes provide a more sensitive measure of regional brain volume differences (for a review see [49]). Overall brain volume was calculated using SIENAX, part of FSL (www.fmrib.ox.ac.uk/fsl/) [50]. It should be noted that for normalized analysis, to be valid, there should be no difference in mean overall brain volume between the patient and control groups.

For completeness, and to make our examination comparable with previous studies, we also carried out a VBM analysis within the hippocampus and amygdala structure. The VBM approach was identical to the whole-brain FSL-VBM analysis but using a binary mask encompassing the amygdala and hippocampus bilaterally. Results were thresholded at t>2.3 and TCFE corrected at p< 0.05.

## Results

### Demographic and clinical data

Demographic data on the patients and controls are shown in Table 1. The two groups were well-matched for age and IQ.

**Table 1:** Demographic and clinic characteristics of patients and controls. All comparisons are two-tailed. Data for IQ were missing from 6 patients and 6 healthy controls (replaced by the group mean). BSL-23 scores were only available for 54 patients.

	Patients (n = 76)	Controls (n = 76)	t-value (d.f.)	P value
**Age (years)**	32.09±6.98	33.55±12.264	0.902 (150)	0.37
**IQ**	97.34 ± 13.90	98.86 ± 10.07	0.768 (144)	0.44
**DIB-R total**	7.82 ± 1.18	-		-
**BSL-23 total**	55.48 ± 25.29	-		-
**Lifetime major depressive disorder present/absent**	39/37	-		-

Thirty-nine of the patients had a lifetime history of major depressive disorder and 37 did not. There were no differences between these two patient groups in age (33.02±6.99 vs 31.10±6.93, t = 1.19, p = 0.23, d.f. = 74), IQ (98.35± 4.20 vs 97.51±15.19, t = 0.25, p = 0.80, d.f. = 74), or in DIB-R (7.79±1.10 vs 7.75±1.25, t = 0.42, p = 0.67, d.f. = 74) and BSL-23 scores (55.40±23.80 vs 34.91±26.01, t = 1.64, p = 0.10, d.f. = 74).

Twenty-four of the BPD patients (31.15%) had no comorbid personality disorder (SCID II data were missing for 6 patients). Twenty-three (30.26%) had one comorbid personality disorder, most frequently showing passive-aggressive, dependent-depressive or paranoid features. Fourteen (18.42%) had two comorbid personality disorders. Eight (10.52%) had three comorbid personality disorders. Two patients (2,60%) of 76 patients had four comorbid personality disorders. Further details are shown in the Supplementary Material, S5 Table.

### Whole-brain VBM analysis

**Fig 1:**
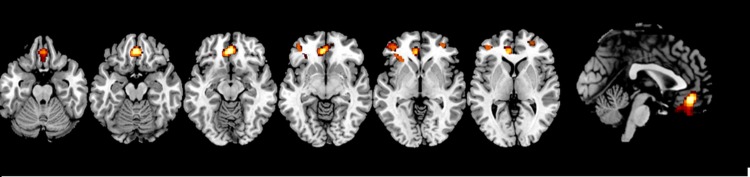
Whole-brain VBM comparison of the BPD patients and controls. Cluster of volume reduction in BPD located in medial frontal cortex and in lateral frontal cortex. The right side of the image is the right side of the brain. Slice labels from left to right z (MNI) = 23 -17 -13 -8 2 4.

The findings are shown in Fig 1 (note: T-test maps are available in neurovault route: https://neurovault.org/images/58125/). Compared to the healthy subjects, the patients with BPD showed three clusters of gray matter reduction, all in the frontal cortex. A cluster of 1014 voxels was seen in the medial frontal cortex, located mostly subgenually, extending from the gyrus rectus to the anterior cingulate gyrus and including part of the orbitofrontal cortex (peak at [MNI: 2, 40, -14], BA 11, t = 4, p = 0.01). There were also two roughly symmetrical clusters in the lateral frontal cortex, (left: 341 voxels peak at [MNI: -38, 46, 4], BA 47, t = 3.12, p = 0.02; right: 66 voxels, peak at [MNI: 30, 50, 2], BA 47, t = 3.61, p = 0.04).

### Relationship of changes found in the whole brain analysis to clinical features

**Fig 2:**
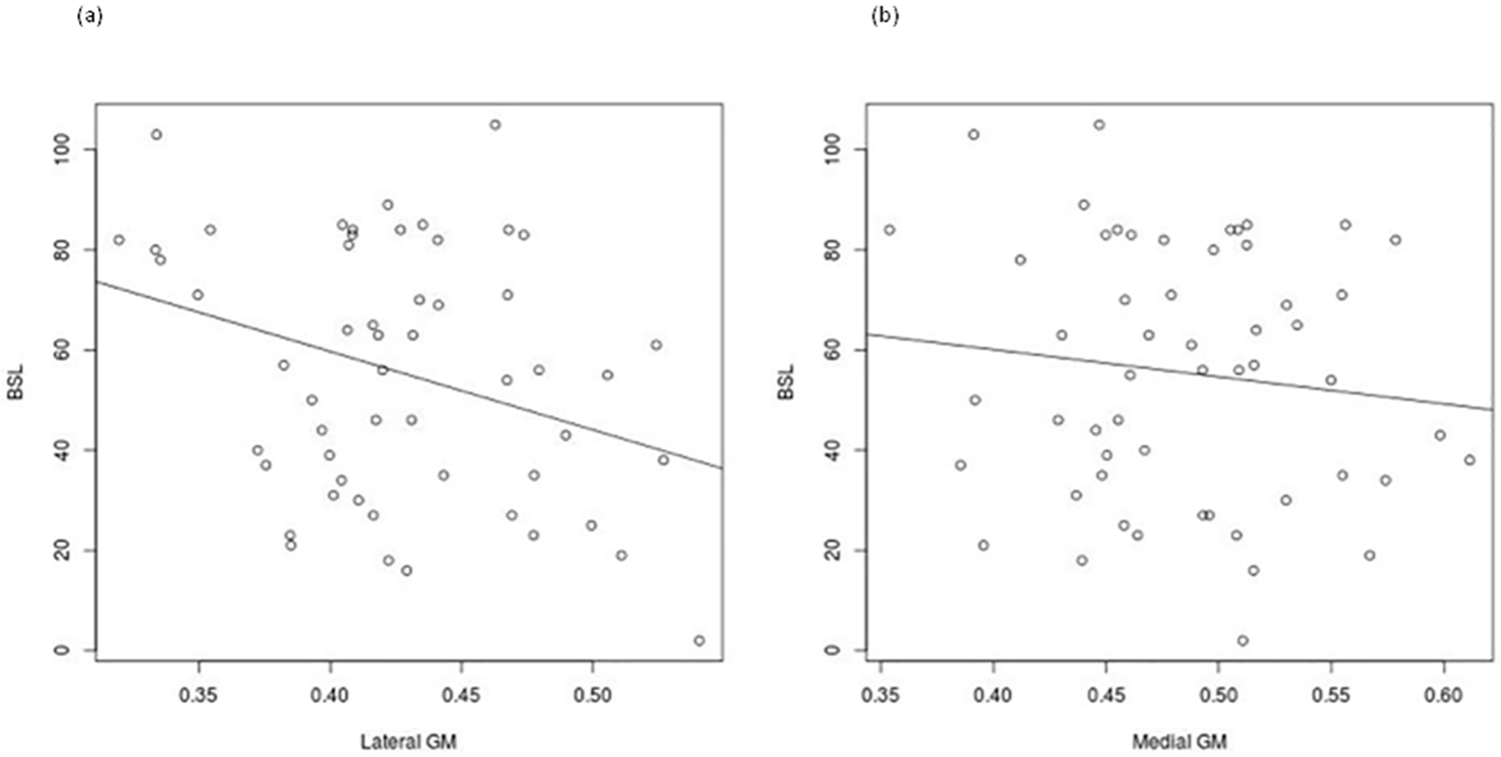
Scatter plots of correlations between: a) mean volume in the combined lateral frontal region and BSL-23 scores, b) mean volume in the medial ROI and BSL-23 scores.

A Shapiro-Wilk normality test was used to verify the normality of distribution of the BSL scores (W = 0.96, p = 0.77) and gray matter volumes in the combined lateral ROI (W = 0.98, p = 0.53) and the medial ROI (W = 0.99, p = 0.87). There was a significant negative correlation between the BSL-23 score and mean volume in the combined lateral frontal ROI (r = -0.32; p = 0.02). This remained significant after controlling for age (r = -0.41, p = 0.002) but became insignificant after controlling for IQ (r = -0.16, p = 0.25). The correlation between BSL-23 score and the medial frontal ROI was not significant. There were no significant correlations between DIB-R total score and mean volumes in either the medial frontal ROI (r = 0.14; p = 0.23) or the combined left and right lateral frontal ROIs (r = -0.06; p = 0.63). Scatter plots are shown in Fig 2.

There were no differences in GM volume between patients with and without history of MDD in either of the two ROIs (medial ROI: MDD mean = 0.48±0.06; no MDD mean = 0.48 ± 0.05; t = -0.03; p = 0.97. combined lateral ROI: MDD mean = 0.42±0.05; no MDD mean = 0.43±0.06; t = 0.70; p = 0.48).

Scatterplots of the mean volumes of the same two ROIs in the 11 patients who were taking antipsychotic medication and the 65 who were not are shown in Supplementary Material, S1 Fig. It can be seen that there is no obvious tendency for the treated patients to have larger or smaller volumes. Given the small number of treated patients statistical analysis was not considered feasible. Individual values are shown in Supplementary Material, S6 Table.

### Hippocampal and amygdala volumes

Absolute volumes for these structures are shown in Table 2. The right amygdala was significantly larger in the patients, but this difference became nonsignificant after FDR correction. All other differences in absolute volumes were nonsignificant.

**Table 2:** Absolute volumes of hippocampus and amygdala (mm^3^) in the patients and controls. All p-values are two-tailed. All d.f. = 150.

Structure	BPD (n = 76)	Controls (n = 76)	t	p	p-FDR corrected
Left amygdala	1044.70±165.21	1043.865±155.91	-0.03	0.97	0.97
Right amygdala	967.17±189.49	907.169±181.73	-1.99	0.048	0.19
Left hippocampus	3425.40±346.53	3510.55±364.28	1.48	0.14	0.28
Right hippocampus	3486.91±403.06	3536.23±356.68	0.80	0.43	0.57

There was no difference in intracranial volume between the two groups (patients 1055784±76261.7 mm^3^ vs controls 1069951±74217.98 mm^3^, t = 1.16; p = 0.25). Comparison of normalized hippocampal and amygdala volumes again revealed enlargement in the right amygdala in the patients, which became nonsignificant after FDR correction (see Supplementary Material, S7 Table).

There were no differences in absolute hippocampus volume between the patients with and without a history of major depression (left: 3451.63±356.74 mm^3^ vs 3397.74± 338.10 mm^3^; t = -0.67, p = 0.50; right: 3509.50±387.47 mm^3^ vs 3463.10± 422.90 mm3; t = -0.50, p = 0.62), or for absolute amygdala volume (left: 1015.79±169.63 mm^3^ vs 1075.16± 156.93 mm3; t = 1.58, p = 0.12; right: 970.86±188.24 mm^3^ vs 963.28 ± 193.32 mm^3^; t = 0.173, p = 0.86).(All d.f. = 74) Results were similar using normalized volumes (see Supplementary Material, S8 Table).

**Fig 3:**
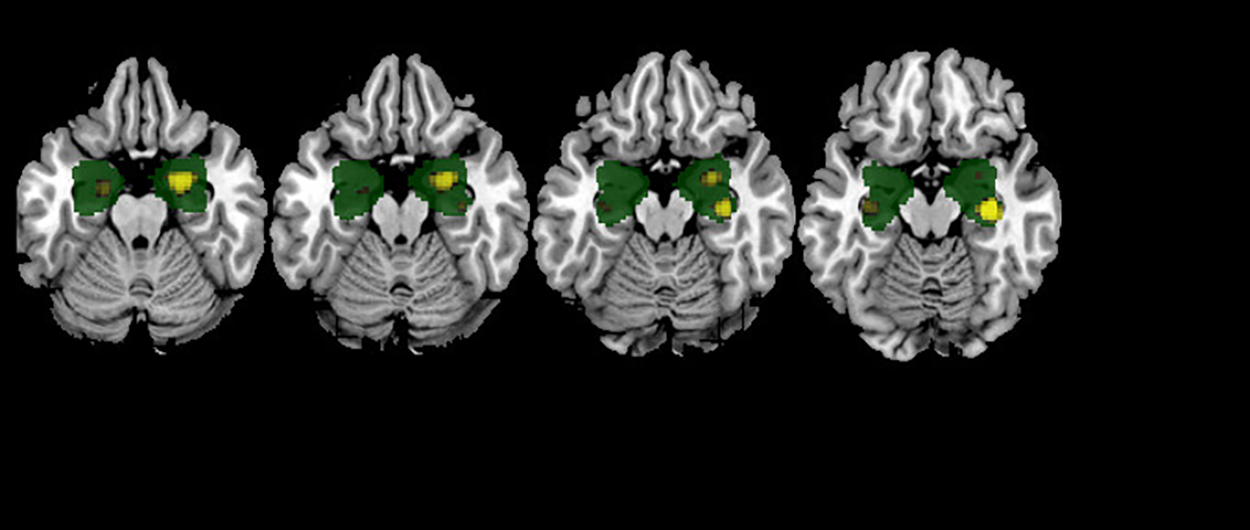
Clusters of increased volume on VBM within the bilateral hippocampus/amygdala mask (mask shown in green). Increased gray matter volume in right amygdala (p = 0.02) and right hippocampus (p = 0.03) is shown in yellow. The right side of the image is the right side of the brain. Slices labels: z (MNI) -22 -20 -18 -16.

VBM analysis masked to the hippocampal and amygdala ROIs revealed that, compared to controls, patients showed clusters of increased gray matter volume in the right hippocampus (120 voxels; peak at [MNI: 32, -22, -12], t = 4.43, p = 0.02) and in the right amygdala (53 voxels; peak at [MNI: 22, -4, -24], t = 3.37, p = 0.03) (see Fig 3; note: T-test maps are available in neurovault route: https://neurovault.org/images/58126/).

Scatterplots of the mean volumes hippocampal and amygdala ROIs in the 11 patients who were taking antipsychotic medication and the 65 who were not are shown in Supplementary Material, S1 Fig. Once again there is no obvious tendency for the treated patients to have larger or smaller volumes. Given the small number of treated patients statistical analysis was not considered feasible. Individual values are shown in Supplementary Material, S6 Table.

## Discussion

The present study, which applied whole-brain VBM with correction to a largest sample of patients and controls, found volume decreases in the dorsolateral prefrontal cortex bilaterally and the ventromedial frontal cortex, particularly the subgenual portion of this. Examination of the hippocampus and amygdala using both conventional volume measurement and VBM failed to confirm previous findings of volume reductions and, if anything, pointed to enlargement. Relationships between volume abnormalities and history of major depression were not seen.

Our finding of volumetric abnormality in the dorsolateral prefrontal cortex could be considered to be consistent with the view that some of the clinical features of BPD, such as impulsiveness and emotional instability reflect frontal lobe dysfunction. Neuropsychological and functional neuroimaging studies of BPD have also been interpreted as supporting a basis for the disorder in altered fronto-limbic function [2,51,52]. However, the neuropsychological findings in BPD have been quite variable, and a meta-analysis did not find any clear evidence for impairment on executive tasks [53]. Functional imaging studies using behavioral inhibition tasks such as the Stroop test and the Go-No Go paradigm have mostly not found reduced prefrontal activations in BPD patients, although this has been demonstrated when an emotional component has been incorporated into the tasks [54]. Our own finding that there was a significant negative correlation between volume in this region and the BSL-23 score, is also open to other interpretations, since it became insignificant when IQ was controlled for, in keeping with studies that have found the volume of this regions to be associated with intelligence [55].

Our study did not find expected volume reductions in the amygdala or hippocampal volume in BPD. The absence of such a finding in the VBM comparison is not unexpected since, for a number of methodological reasons, whole-brain VBM is less sensitive to volume alterations in small subcortical structures than large cortical areas (eg see [56,57]). However, volume reductions in these structures were also not seen when ROI analysis were employed, which instead found evidence for an increase in the right amygdala volume in the BPD patients (although only significantly without correction for multiple comparisons). This finding goes against two meta-analyses of ROI studies [14,15]. The reason or reasons for this discrepancy are obscure, although the fact that our study was considerably larger than the studies contributing to the two ROI-based meta-analysis might be important. VBM analysis masked to the hippocampus and amygdala also tended to support a finding of right-sided enlargement in both structures. This was found to be restricted to a small area in both structures, something that could reflect either a genuine localization of the enlargement, or alternatively could simply be where a generalized enlargement was most marked and became suprathreshold.

There was little to suggest from our study that brain volume reductions in BPD are explainable in terms of co-existent major depression, which is itself associated with cortical and hippocampal volume reductions [33]. Nevertheless, the pattern of cortical volume reductions we found is not dissimilar to that seen in major depression. Thus, a meta-analysis of VBM studies of major depressive disorder by Bora et al [25] found a single volume of volume reduction in the medial frontal cortex. This was located more rostrally and closer to the genu of the corpus callosum than the corresponding cluster in our BPD patients. When a less conservative threshold was used, this cluster extended more rostrally and also into the subgenual anterior cingulate cortex. An additional cluster of volume reduction in the right dorsolateral frontal cortex also appeared at this lower threshold.

One possible interpretation of this similarity is that the some of the brain structures affected in both BPD and affective disorder are involved in the normal regulations of mood. Of relevance here is a theoretical review by Price and Drevets [58]: they noted that the medial frontal/orbitofrontal cortex has anatomical connections to the amygdala, ventral striatum and the hypothalamus, and has also been found to show functional imaging changes in both major depression and normal sadness. Accordingly, they argued that this cortical region is involved in ‘forebrain modulation of visceral function in response to sensory or emotive stimuli’, something that could easily translate into a role in emotional and mood disorders in humans.

Some limitations of our study need to be acknowledged. First, some patients with BPD were taking antipsychotic and antidepressant medication, something that has the potential to affect brain structure, at least in the case of antipsychotics. Secondly, BPD is associated with other comorbidities besides major affective disorder which may affect brain structure, including alcohol/drug abuse [59,60] and also perhaps post-traumatic stress disorder, although this last association is currently undecided [11]. We only excluded substance abuse within the six prior months to participation but we did not examine patients for history of post-traumatic stress disorder. Finally, in contrast to some but not all VBM studies of BPD, the healthy controls in this study were not screened for presence of axis II disorders, ie personality disorders. This might account for some of the heterogeneity among the findings to date.

## Supporting information

S1 Table Sociodemographic and clinical data from borderline subjects and healthy subjets. (XLSX)

S2 Table Amygdala and hippocampal volumes from borderline subjects and healthy subjects (mm3 and corrected). (XLSX)

S3 Table Cluster characteristics in the whole-brain VBM comparison between borderline subjects and healthy subjects. (DOCX)

S4 Table Relationship of changes found in the whole brain analysis to clinical features: BSL-23 and DIB-R scores. As seen in article, there was a significant negative correlation between the BSL-23 score and mean volume in the combined lateral frontal ROI (r = -0.32; p = 0.02). This remained significant after controlling for age (r = -0.41, p = 0.002) but became insignificant after controlling for IQ (r = -0.16, p = 0.25). The correlation between BSL-23 score and the medial frontal ROI was not significant. There were no significant correlations between DIB-R total score and mean volumes in either the medial frontal ROI (r = 0.14; p = 0.23) or the combined left and right lateral frontal ROIs (r = -0.06; p = 0.63).
(XLSX)

S5 Table Commorbid personality disorders in the sample of Borderline Personality Disorder patients. As seen in article, 31,15% of patients had no comorbid personality disorder. We summarize all data for each patient. (SCID II data were missing for 6 patients).
(XLSX)

S6 Table Individual data of Grey Matter volume of hippocampus, amygdala and ROIs in patients with and without antipsychotics. (XLSX)

S7 Table Normalized volumes of hippocampus and amygdala in the patients and controls. (DOCX)

S8 Table Normalized volumes of hippocampus and amygdala in the patients with and without Major Depressive Disorder (MDD) history. (DOCX)

S9 Table Volumes of Grey Matter (GM), White Matter (WM), Cerebro Spinal Fluid (CSF) and Intracranial Total Volume (ICTV) in Borderline patients and controls. (XLS)

S1 Fig Scatterplots of amygdala, hippocampus, Lateral ROI and Medial ROI volumes: Comparison between patients with and without antipsychotic treatment. (PDF)
