# Supplementary material for: Abnormalities in gray matter volume in patients with borderline personality disorder and their relation to lifetime depression: A VBM study
Source: PLoS One. 2018 Feb 21;13(2):e0191946. doi: 10.1371/journal.pone.0191946 (PMC5842882; doi:10.1371/journal.pone.0191946)
Supplement: S3 Table — (DOCX) [file pone.0191946.s003.docx]

**S3 Table. Cluster characteristics in the whole-brain VBM comparison between borderline subjects and healthy subjects**

| **Cluster** | **Voxels** | **p** | **x_MAX (mm)** | **y_MAX (mm)** | **z_MAX (mm)** | **x_COG (mm)** | **y_COG (mm)** | **z_COG(mm)** |
| --- | --- | --- | --- | --- | --- | --- | --- | --- |
| 4 | 1014 | 0.011 | 2 | 40 | -14 | -2.19 | 38.8 | -12.6 |
| 3 | 341 | 0.023 | -38 | 46 | -4 | -38.6 | 42.1 | -2.52 |
| 2 | 66 | 0.043 | 30 | 50 | 2 | 30.8 | 51.1 | 1.18 |
| 1 | 3 | 0.05 | 14 | 56 | 2 | 14 | 55.3 | 0.667 |
