# Supplementary material for: Abnormalities in gray matter volume in patients with borderline personality disorder and their relation to lifetime depression: A VBM study
Source: PLoS One. 2018 Feb 21;13(2):e0191946. doi: 10.1371/journal.pone.0191946 (PMC5842882; doi:10.1371/journal.pone.0191946)
Supplement: S7 Table — (DOCX) [file pone.0191946.s007.docx]

| **Structure** | **BPD (n=76)** | **Controls (n=76)** | **t-value** | **p-value** | **p-FDR corrected** | **Cohen’s d estimate** |
| --- | --- | --- | --- | --- | --- | --- |
| Left amygdala | 0.00099±0.00015 | 0.00097±0.00013 | 0.63 | 0.5282 | 0.72 | -4.22589 |
| Right amygdala | 0.00092±0.00018 | 0.00085±0.00017 | 2.50 | 0.014 | 0.05 | -4.22617 |
| Left hippocampus | 0.0033±0.00033 | 0.0033±0.0003 | 0.61 | 0.54 | 0.72 | -4.219445 |
| Right hippocampus | 0.0033±0.0004 | 0.0033±0.00032 | 0.02 | 0.984 | 0.98 | -4.219325 |
