# Supplementary material for: Abnormalities in gray matter volume in patients with borderline personality disorder and their relation to lifetime depression: A VBM study
Source: PLoS One. 2018 Feb 21;13(2):e0191946. doi: 10.1371/journal.pone.0191946 (PMC5842882; doi:10.1371/journal.pone.0191946)
Supplement: S8 Table — (DOCX) [file pone.0191946.s008.docx]

| **Structure** | **BPD with MDD(n=39)** | **BPD without MDD (n=37)** | **t-value** | **p-value** | **p-FDR corrected** | **Cohen’s d estimate** |
| --- | --- | --- | --- | --- | --- | --- |
| Left amygdala | 0.00099±0.00018 | 0.00098±0.00014 | 0.92 | 0.36 | 0.72 | 0.2114082 |
| Right amygdala | 0.00092±0.000153 | 0.0009342±0.00017 | -0.81 | 0.42 | 0.09 | -0.185122 |
| Left hippocampus | 0.00318±0.000348 | 0.00332±0.0003033 | -1.89 | 0.06 | 0.82 | -0.43723 |
| Right hippocampus | 0.00324±0.000411 | 0.00338±0.00034 | -1.6 | 0.11 | 0.88 | -0.3701803 |

BPD: Borderline Personality Disorder

MDD: Major Depressive Disorder
