# Supplementary material for: Abnormalities in gray matter volume in patients with borderline personality disorder and their relation to lifetime depression: A VBM study
Source: PLoS One. 2018 Feb 21;13(2):e0191946. doi: 10.1371/journal.pone.0191946 (PMC5842882; doi:10.1371/journal.pone.0191946)
Supplement: S1 Fig — (PDF) [file pone.0191946.s010.pdf]

Scatterplot of amygdala volumes: Borderline patients with antipsychotic therapy vs Borderline patients without antipsychotic therapy.

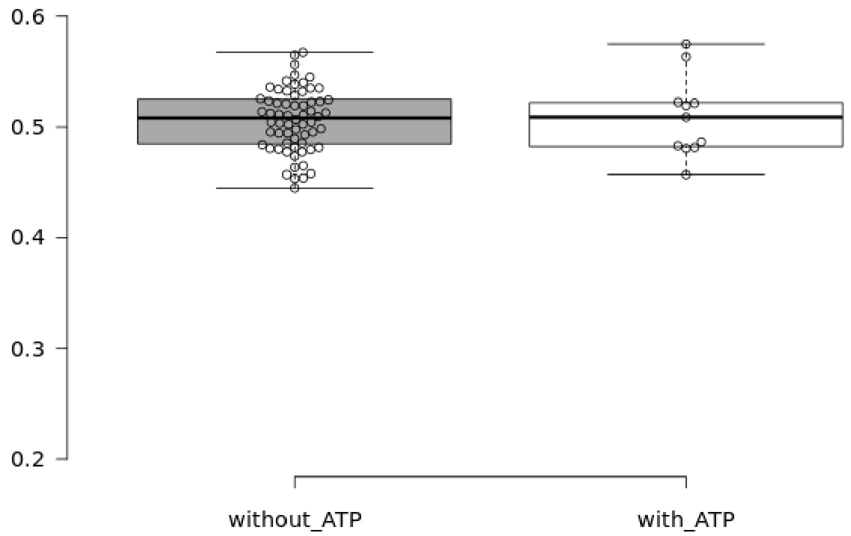

Scatterplot of hippocampus volumes: Borderline patients with antipsychotic therapy vs Borderline patients without antipsychotic therapy.

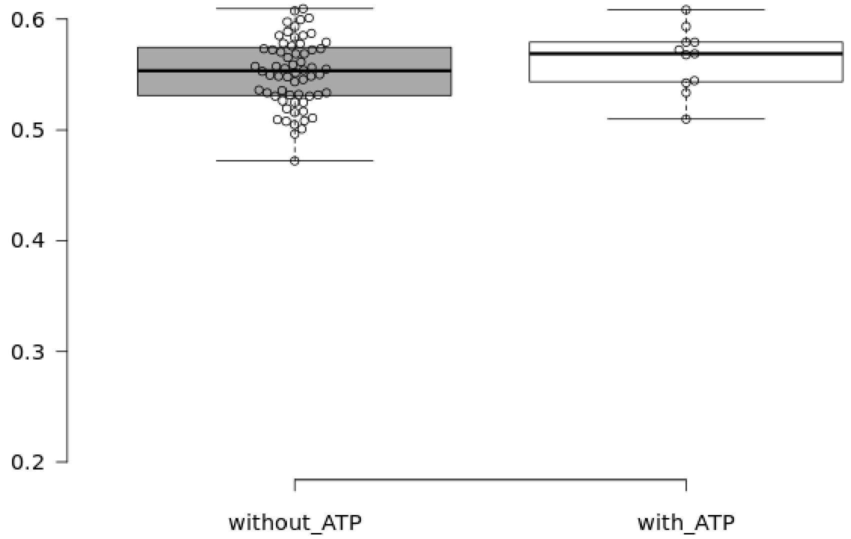

Scatterplot of Lateral ROI volume: Borderline patients with antipsychotic therapy vs Borderline patients without antipsychotic therapy.

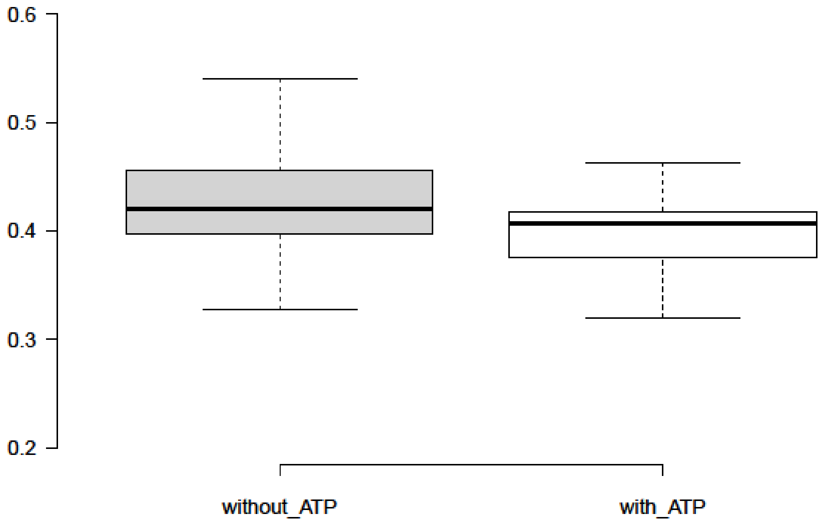

Scatterplot of Medial ROI volume: Borderline patients with antipsychotic therapy vs Borderline patients without antipsychotic therapy.

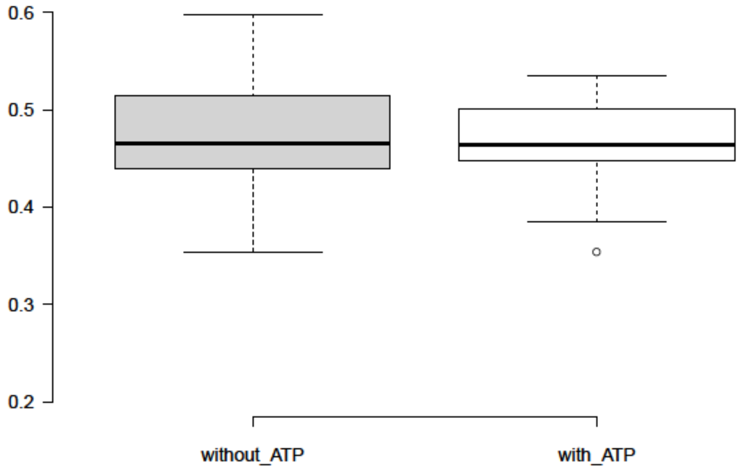
